# General Œdema after the Use of Quinine

**Published:** 1846-11

**Authors:** 


### GENERAL ŒDEMA AFTER THE USE OF QUININE.

Dr. Henry Strong, of Mississippi, a short time since, communicated to us the following facts, at the same time enclosing a specimen of the suspected sulphate of quinine, concerning the purity of which he requested our opinion. On the application of the usual tests no adulteration of the article was discovered:

A planter of this vicinity purchased a bottle of sulphate of quinine from an apothecary in Woodville. Before using it he offered it to me for inspection; it was very lumpy, and some of the lumps were of a color nearly as yellow as sulphur. I remarked to him that it had not the usual appearance, and was probably an inferior article. He opened the bottle and gave a portion of the medicine to four or five negroes who had been laboring under common bilious remitting fever. They seemed to convalesce rapidly, but in three or four days they came in swelled severely in the head, body, and extremities. The distention of the abdomen and lower extremities seemed to be as great as the skin could bear. This swelling subsided gradually in about a week. From the fact that all who took of the quinine were swelled, while no others on the plantation were similarly affected, suspicion falls naturally on that medicine.

September 17th, 1846.

